# Metal–organic framework (UiO-66 and UiO-66-NH_2_)-based adsorbents for bilirubin removal used in hemoperfusion†

**DOI:** 10.1039/d3ra07212f

**Published:** 2023-12-01

**Authors:** Yi Liu, Zhipeng Yuan, Yanrong Chen

**Affiliations:** a Department of Traditional Chinese Medicine, Jinan Fourth People's Hospital Jinan Shandong PR China; b Shandong Key Laboratory for Special Silicon-containing Material, Advanced Materials Institute, Qilu University of Technology (Shandong Academy of Sciences) Jinan Shandong PR China yuanzp@sdas.org; c The Affilited Hospital of Shandong University of Traditional Chinese Medicine Jinan Shandong PR China xiaopang-kk@163.com

## Abstract

Excessive accumulation of bilirubin in patients with hyperbilirubinemia can lead to tissue and organ damage and neurological diseases, and is even life-threatening in severe cases. Hemoperfusion is an effective method for removing bilirubin, but clinically used hemoperfusion adsorbents have unsatisfactory adsorption capacity and kinetics. In order to obtain a safe and efficient bilirubin adsorbent, Zr-based Metal–Organic Framework (MOF) material UiO-66 with high specific surface area and aqueous medium stability was prepared and modified with varying degrees of amination to improve its adsorption capacity. According to adsorption experiments in aqueous solution and simulated plasma, it was confirmed that the unsaturated coordinated zirconium in UiO-66 can effectively induce the aggregation and precipitation of free bilirubin unbound to albumin and the amino group on UiO-66-NH_2_ has a strong affinity for albumin bound bilirubin. The adsorption effect of UiO-66-NH_2_ with a high degree of amino modification is significantly stronger than that of UiO-66-NH_2_ with a low degree of modification. In simulated plasma with a bilirubin concentration of 40 mg dL^−1^, the adsorption capacities of UiO-66 and UiO-66-NH_2_-1.9 can reach 69.08 mg g^−1^ and 81.13 mg g^−1^. The adsorption isotherm fitting and adsorption kinetics fitting results also show that UiO-66 and UiO-66-NH_2_ are good adsorbents for bilirubin. In dynamic adsorption, the adsorbents also showed good performance and did not affect the protein in the plasma. The hemolysis test, coagulation time test, and cytotoxicity test confirmed that the bilirubin adsorbents based on UiO-66 and UiO-66-NH_2_ have good blood compatibility and biocompatibility. This study provides new ideas for the development of a novel bilirubin adsorbent and a theoretical basis for the study of bilirubin adsorption mechanisms.

## Abstract 2

UiO-66 and amino modified UiO-66-NH_2_ hemoperfusion adsorbents were prepared. The unsaturated coordination zirconium and amino functional groups on UiO-66-NH_2_ can achieve efficient removal of albumin bound bilirubin.

## Graphical abstract

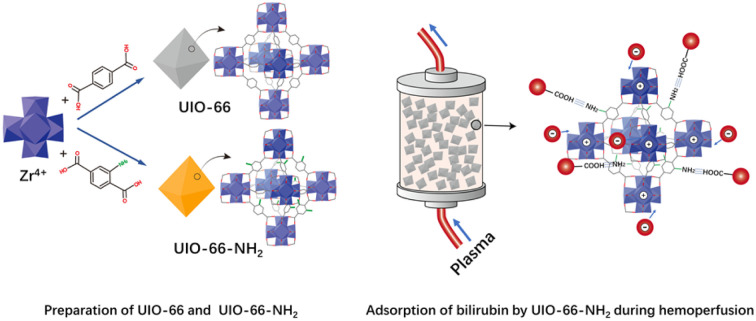

## 1. Introduction

Bilirubin is a metabolic product formed by the normal breakdown and metabolism of hemoglobin in aging red blood cells. It has a high affinity with albumin and usually binds to albumin in the blood to form water-soluble complexes, which are transported to the liver for excretion.^1,2^ In the case of liver damage or metabolic obstruction, the concentration of bilirubin in the body will sharply increase, known as hyperbilirubinemia. Excessive accumulation of bilirubin in the body can cause yellow discoloration of skin, tissue and organ damage, neurological diseases, and even endanger life in severe cases.^3^ In clinical practice, methods such as plasma exchange (PEX), hemodialysis (HD) and hemoperfusion (HP) have been widely used to treat hyperbilirubinemia.^4,5^ Among these methods, PEX therapy requires large volumes of fresh frozen plasma, which is expensive and difficult to obtain. Hemodialysis has a poor removal effect on albumin bound bilirubin, and HP is the most effective method for treating hyperbilirubinemia.^6,7^

The adsorbent in the HP column determines the effectiveness of bilirubin removal, so the development of safe and efficient bilirubin adsorbents is of great significance.^8^ Traditional bilirubin adsorbents such as activated carbon,^9^ adsorption resin,^10^ polymer beads^11^ and bilirubin imprinted polymers^12^ and novel bilirubin adsorbents based on nanomaterials including aerogels,^13,14^ MOFs,^15,16^ nanofiber materials,^3,17^ MXene^18,19^ have been continuously reported and innovated for many years to improve adsorption efficiency. Among them, MOFs material is one of the most attractive adsorption materials. MOFs are a promising class of crystalline porous materials composed of inorganic nodes and organic linkers. They have high porosity and specific surface area, clear pore size, and functional pore environment, which make them widely used in many fields.^20–22^ These attractive characteristics also endow MOFs with strong adsorption capacity and have great potential for application as adsorbents in HP. Due to direct contact with plasma, the chemical stability of MOFs in aqueous media is crucial. UiO-66 is one kind of Zr-MOFs that exhibits high chemical stability in aqueous media. It has been widely used in adsorption,^23^ catalysis,^24^ and drug delivery^25^ in the aqueous phase. The adsorption mechanisms, such as electrostatic interactions, hydrogen bonding and p–p interaction have also been extensively studied. Therefore, UiO-66 has great potential as a safe and efficient bilirubin adsorbent.

In addition to the advantage of large specific surface area, unsaturated coordination zirconium of UiO-66 also has affinity for carboxyl groups on bilirubin. In addition, it can further improve adsorption capacity through functionalized modification. Bilirubin is negatively charged, so the amination modification of materials is a commonly used functionalization method in the preparation of bilirubin adsorbents.^17^ Amine groups can tightly bind to the carboxyl group (–COOH) within bilirubin molecules through electrostatic interactions. It has been proven that increasing the density of amino groups in membrane materials can improve adsorption efficiency,^26^ which inspired us to introduce amino groups on the surface of UiO-66. It may become an effective pathway to improve its bilirubin adsorption capacity, but further research is needed on its specific effects and mechanisms.

In this work, UiO-66 adsorbent and UiO-66-NH_2_-1, UiO-66-NH_2_-1.45 and UiO-66-NH_2_-1.9 adsorbents obtained by varying degrees of amino modification of UiO-66 for the removal of bilirubin from hyperbilirubinemia plasma were prepared. Bilirubin molecules are negatively charged in aqueous solutions, and the unsaturated coordination zirconium on UiO-66 is electropositive.^27^ Therefore, UiO-66 adsorbent has a strong adsorption effect on free bilirubin in aqueous solution. Moreover, due to charge interference with the dissolution equilibrium, UiO-66 can also induce aggregation and precision of bilirubin, and the effect removing bilirubin of UiO-66 in aqueous solution is better than UiO-66-NH_2_. In simulated plasma, bilirubin binds stably to albumin, amino groups on UiO-66-NH_2_ have stronger adsorption effect on it.^17^ Meanwhile, the adsorption effect of UiO-66-NH_2_ with high degree of amino modification is significantly stronger than that of UiO-66-NH_2_ with low degree of amino modification. The fitting results of adsorption isotherms and adsorption kinetics also confirms these conclusions. All UiO-66 and UiO-66-NH_2_ adsorbents have good biocompatibility and blood compatibility. In dynamic adsorption experiments, the adsorbents also exhibit good performance and do not adsorb proteins in plasma, making them suitable for removing bilirubin through HP. This study provides new ideas for the development of novel bilirubin adsorbent and theoretical basis for the study of bilirubin adsorption mechanisms.

## 2. Materials and methods

### 2.1 Materials

Zirconium oxychloride octahydrate, 2-aminoterephthalic acid, terephthalic acid, acetic acid, hydrochloric acid are all purchased from Aladdin Bio-Chem Technology Co. Ltd. (China); bilirubin, *N*,*N*-dimethylformamide (DMF, ≥99%), bovine serum albumin (BSA, ≥98%) are purchased from Innochem Technology Co. Ltd. Enhanced BCA protein assay kit and cell counting kit-8 (CCK-8) were from Beyotime Institute of Biotechnology (China). Prothrombin Time (PT) assay kit and Activated Partial Thromboplastin Time (APTT) assay kit are from Shanghai Sun Biotechnology Co., Ltd. The plasma comes from volunteer (male, 32 years old). All buffers were prepared with ultra-pure Milli-Q water (resistance > 18 MΩ cm^−1^).

### 2.2 Preparation of UiO-66 and UiO-66-NH_2_

To prepare UiO-66, 6 g (36.12 mmol) terephthalic acid and 8.03 g (24.92 mmol) zirconium oxychloride octahydrate were dissolved in 150 mL DMF, under vigorous stirring, 1.5 mL of hydrochloric acid (37%) and 2 mL of acetic acid were added to the solution, and continue to stir for 5 min. Then, the obtained solution was sealed and heated to 100 °C for two hours. It can be observed that the solution has become gel. The gel was broken into small pieces, washed with 100 mL DMF and centrifuged, washed with ethanol three times and centrifuged. After that, the crushed gel was put into water for solvent replacement, and the water was replaced every 12 hours for three days. Finally, the crushed gel was freeze-dried to obtain UiO-66 powder.

The preparation method of UiO-66-NH_2_-*X* (*X* = 1, 1.45 and 1.9, which represents the 2-aminoterephthalic acid/Zr molar ratio) was the same as that of UiO-66 except that the ligand terephthalic acid was replaced by 2-aminoterephthalic acid and the ligand/metal molar ratio was adjusted and they are respectively named as UiO-66-NH_2_-1, UiO-66-NH_2_-1.45 and UiO-66-NH_2_-1.9. For consistency, the UiO-66 with terephthalic acid/Zr molar ratio 1 : 1 is named as UiO-66-1. The material preparation method referred to previously reported literature.^28^

### 2.3 Characterization

The morphologies of UiO-66-1 and UiO-66-NH_2_-*X* were examined by scanning electron microscope (SEM, Zeiss Supra 55, Germany). The crystal structure was characterized by X-ray diffraction (XRD, Bruker, D8 Advance, Germany). Fourier-transform infrared (FTIR) spectra were taken on a PerkinElmer Spectrum 100 apparatus (America) in the frequency range from 4000 to 500 cm^−1^.

### 2.4 Adsorption isotherm

To make the adsorption isotherm of the samples towards bilirubin, UiO-66-1, UiO-66-NH_2_-1, UiO-66-NH_2_-1.45 and UiO-66-NH_2_-1.9 were all weighted for two doses of 30 mg and they were respectively dispersed into 10 mL bilirubin aqueous solution and bilirubin loaded BSA solution (simulated plasma) with different bilirubin concentrations (5, 10, 15, 20, 30 and 40 mg dL^−1^). The preparation method of bilirubin aqueous solution is to dissolve the weighed bilirubin in a small amount of 0.1 mol per L NaOH solution, transfer it to a volumetric flask, and dilute it to the corresponding scale with 0.05 mol per L phosphate buffer (pH = 7.2–7.5). The preparation method of bilirubin loaded BSA solution (simulated plasma) is similar, except that an appropriate amount of BSA needs to be added to the prepared aqueous solution to achieve a BSA concentration of 40 g L^−1^. The mixture was shaken in a shaker at a speed of 150 rpm at room temperature (25 °C), after adsorption for 4 h, the concentrations of bilirubin in aqueous solution and simulated plasma were determined by measuring the absorbance of the two kind solutions at 438 nm and 452 nm respectively by UV-vis spectrometer. It should be emphasized that the absorption value of the binding compound between bilirubin and albumin at 452 nm will not be affected by the isosbestic point (416 nm).^29^ All simulated plasma used in this article are 40 g per L albumin aqueous solutions. The equilibrium adsorption amounts *Q*_e_ (mg g^−1^) were calculated with the following equation:1
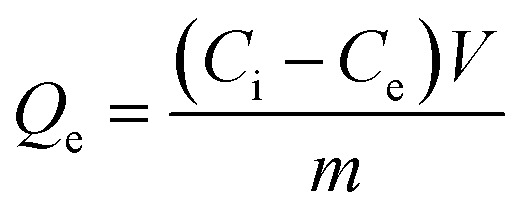
where *C*_i_ (mg mL^−1^) and *C*_e_ (mg mL^−1^) are the initial and equilibrium bilirubin concentration respectively. *V* (mL) is the volume of solution and *m* (g) is the mass of the sample.

### 2.5 Adsorption kinetics

To investigate the adsorption kinetics of relevant samples to bilirubin, UiO-66-1, UiO-66-NH_2_-1, UiO-66-NH_2_-1.45 and UiO-66-NH_2_-1.9 were all weighted for two doses of 30 mg and respectively dispersed into 10 mL bilirubin aqueous solution (30 mg dL^−1^) and bilirubin loaded simulated plasma (30 mg dL^−1^), then shake the mixture on a shaker at a speed of 150 rpm for 20, 40, 60, 80, 100, 120 min at room temperature. At each set time point, the concentrations of bilirubin were determined and the bilirubin adsorption amounts were calculated with eqn (1).

### 2.6 Dynamic adsorption of bilirubin

The dynamic adsorption tests were performed with a peristaltic pump and a laboratory made small HP device. The inner diameter of the blood perfusion column is 22 mm and the length is 90 mm. The two ends of the column are connected to the two ends of a silicone tubing. 120 mg of UiO-66-1, UiO-66-NH_2_-1, UiO-66-NH_2_-1.45 and UiO-66-NH_2_-1.9 samples are placed in the column in sequence with filter paper placed at both ends of the column to prevent the adsorbent from entering the silicone tubing. 40 mL of the bilirubin aqueous solution or bilirubin loaded simulated plasma with bilirubin concentration of 30 mg dL^−1^ is injected into the column and the silicone tubing is clamped into the peristaltic pump. Adjust the rotational speed of the peristaltic pump to achieve a liquid flow rate of 1 mL min^−1^, so that the liquid can circulate in a closed loop formed by the silicone tubing and HP column to ensure sufficient adsorption. Dynamic adsorption is carried out at room temperature (25 °C), and the up-flow way is selected to pass through the column. After 0 min, 20 min, 40 min, 60 min, 80 min, 100 min, 120 min circulation, the bilirubin concentrations were determined, meanwhile, the total protein concentration of the bilirubin loaded simulated plasma were also determined by enhanced BCA protein assay kit.

### 2.7 Coagulation times and hemolysis assay

PT (prothrombin time) and APTT (active partial thromboplastin time) were determined by Prothrombin Time Assay Kit and Activated Partial Thromboplastin Time Assay Kit and the operation steps were accurately carried out according to the instructions. Proper amount of UiO-66-1, UiO-66-NH_2_-1, UiO-66-NH_2_-1.45 and UiO-66-NH_2_-1.9 were placed in clean centrifuge tubes, and the platelet poor plasma (PPP; obtained by centrifuging blood at 3000 rpm for 15 min) was used to incubate with the samples at 37 °C for 0.5 h. 50 μL of the PPP was taken for PT and APTT tests each time.

In hemolysis assay, UiO-66-1, UiO-66-NH_2_-1, UiO-66-NH_2_-1.45 and UiO-66-NH_2_-1.9 were incubated in the resuspended red blood cell (RBC) solution containing 2% RBCs and 98% PBS at 37 °C for 30 min. The solution containing 2% RBCs and 98% PBS was used as negative control and the solution containing 2% RBCs and 98% pure water was used as positive control. After incubation, all samples were centrifuged at 700 g (2710 rpm) for 10 min and were photographed. The absorbance of the supernatant was measured at 541 nm, and the hemolysis ratio (HR) was calculated by the following equation:2
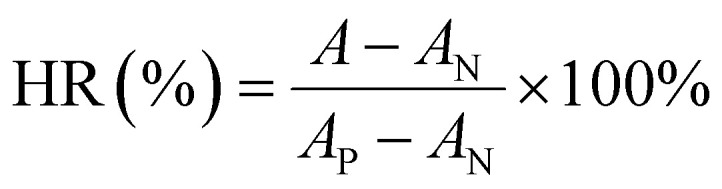
where *A*, *A*_N_, and *A*_P_ are the absorbance of the samples, negative control and positive control, respectively. All experiments were repeated three times.

### 2.8 *In vitro* cytocompatibility assay

Mouse 3T3 fibroblasts cells (ATCC) were cultured in DMEM (Hyclone, USA) supplemented with 5% FBS. Cells were maintained in a 5% carbon dioxide incubator at 37 °C. For cytocompatibility assay, all samples were decontaminated under UV light for 2 h and 1 × 10^4^ 3T3 cells were cocultured with the samples for 6 h, 12 h and 24 h, cell viability was measured using CCK-8.

## 3. Results and discussion

### 3.1 Preparation and characterization of UiO-66-1 adsorbent and amino modified UiO-66-1 adsorbent

**Fig. 1:**
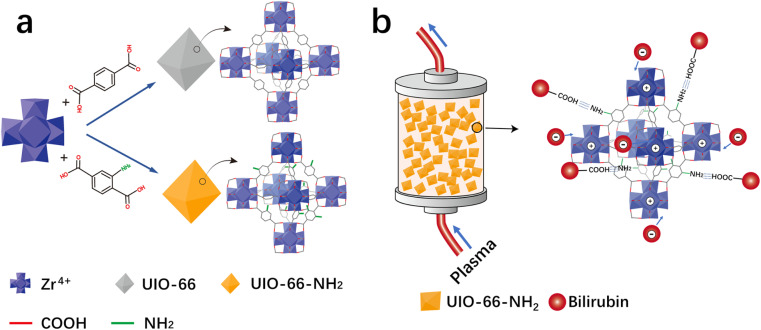
Schematic illustration showing (a) the structures of UiO-66-1 and UiO-66-NH_2_ and (b) HP process.

**Fig. 2:**
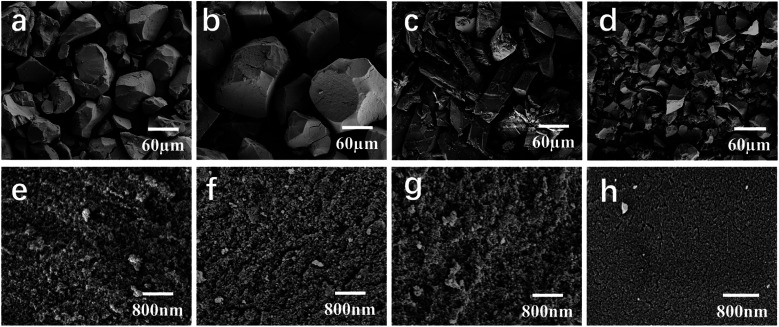
SEM images showing the representative morphological structures of UiO-66-1 in (a) low and (e) high magnifications, UiO-66-NH_2_-1 in (b) low and (f) high magnifications, UiO-66-NH_2_-1.45 in (c) low and (g) high magnifications and UiO-66-NH_2_-1.9 in (d) low and (h) high magnifications.

The adsorption capacity of HP adsorbent is closely related to its microstructure and surface functional groups. In order to introduce amino groups on UiO-66, as shown in Fig. 1a, UiO-66 was modified by changing the ligand. The adsorption effect of UiO-66 adsorbent mainly relies on the electrostatic attraction of positive charges on unsaturated coordination zirconium to attract negatively charged bilirubin. For the UiO-66-NH_2_, in addition to the adsorption effect of unsaturated coordination zirconium, its surface amino groups also have an attractive effect on the carboxyl groups on bilirubin, which is shown in Fig. 1b. The degree of amino modification is achieved by adjusting the ligand ratio. The morphology diagrams of UiO-66-1, UiO-66-NH_2_-1, UiO-66-NH_2_-1.45, and UiO-66-NH_2_-1.9 with different degrees of amino modification are shown in Fig. 2. From Fig. 2a, it can be seen that UiO-66-1 has a sharp block structure. In Fig. 2b, the block size of UiO-66-NH_2_-1 with 2-aminoterephthalic acid as the ligand is larger, which may be due to different ligands causing different crystallinity of the materials. In Fig. 2c and d, the block size of UiO-66-NH_2_-1.45 and UiO-66-NH_2_-1.9 gradually decreases compared to UiO-66-NH_2_-1, this may be due to an increase in ligand ratio, resulting in a decrease in crystal degree.

**Fig. 3:**
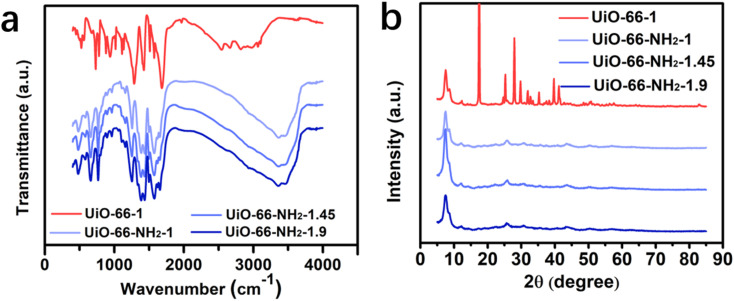
Characterization of UiO-66-1 and UiO-66-NH_2_. (a) FTIR spectrums and (b) X-ray diffraction patterns of UiO-66-1, UiO-66-NH_2_-1, UiO-66-NH_2_-1.45 and UiO-66-NH_2_-1.9.

To confirm the amino modification of UiO-66, the infrared spectra before and after modification were tested. As shown in Fig. 3a, a large and broad absorption peak around 3421 cm^−1^ can be found on the spectra of UiO-66-NH_2_-1, UiO-66-NH_2_-1.45 and UiO-66-NH_2_-1.9, but not on the spectra of UiO-66-1, which can be attributed to the coincidence of the symmetric and asymmetric vibration peaks of –NH_2_ at 3362 cm^−1^ and 3464 cm^−1^. This indicates the successful introduction of amino groups on the surface of UiO-66-1 by modifying the ligand. The peak at 1583 cm^−1^ on all spectra was the characteristic peak of C=O bond in the carboxyl group. The peaks at 800–600 cm^−1^ can be attributed to the group of Zr–O. The crystal structure of UiO-66-NH_2_ was determined through XRD analysis. As shown in Fig. 3b. The diffraction peaks in the spectrum near 7.48°, 8.59°, 12.09°, 25.61° and 32.96° corresponding to (111), (002), (022), (224) and (137) crystal planes, indicating the successful synthesis of UiO-66-NH_2_ with atoms exhibit in a face-centered cubic configuration.

### 3.2 Adsorption isotherm

**Fig. 4:**
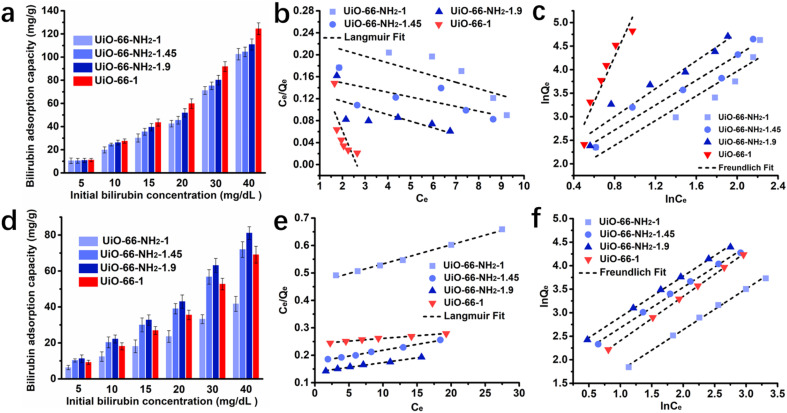
Bilirubin adsorption performance of the samples in aqueous solution, (a) adsorption capacity, fitting results of (b) Langmuir isotherms and (c) Freundlich isotherms of UiO-66-1, UiO-66-NH_2_-1, UiO-66-NH_2_-1.45 and UiO-66-NH_2_-1.9; bilirubin adsorption performance of the samples in bilirubin loaded simulated plasma, (d) adsorption capacity, fitting results of (e) Langmuir isotherms and (f) Freundlich isotherms of UiO-66-1, UiO-66-NH_2_-1, UiO-66-NH_2_-1.45 and UiO-66-NH_2_-1.9.

To understand the adsorption mechanism better, the maximum adsorption capacities and adsorption isotherms were assessed. The adsorption capacities at different initial concentrations of UiO-66-1, UiO-66-NH_2_-1, UiO-66-NH_2_-1.45 and UiO-66-NH_2_-1.9 in aqueous solution and simulated plasma are shown in Fig. 4a, d, Tables S1 and S2 in ESI.† The equilibrium adsorption capacity of all samples increased with the bilirubin initial concentration in aqueous solution and UiO-66-1 had the maximum adsorption capacity among these adsorbents. This indicates that the zirconium on UiO-66-1 has a strong adsorption effect on free bilirubin, and the presence of amino groups does not further improve the adsorption force, but rather produces a steric hindrance effect that affects the adsorption of bilirubin. In simulated plasma, the adsorption capacity of both UiO-66-NH_2_-1.45, UiO-66-NH_2_-1.9 exceeds UiO-66-1, indicating that the adsorption capacity of amino groups for albumin bound bilirubin is relatively stronger. As the proportion of amino groups increased, the adsorption capacity also increases.

The adsorption isotherm results were fitted by two widely used models named Langmuir and Freundlich isotherm models. The Langmuir isotherm model was suitable for monolayer adsorption onto a surface with a finite number of identical sites, and the Freundlich isotherm model was used for heterogeneous systems with multilayer adsorption. The two isotherm models were mathematically described as follows:3
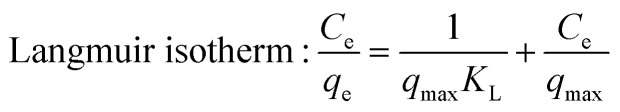
4
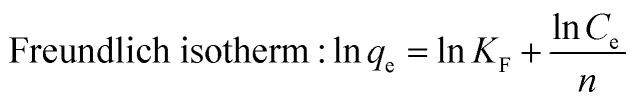
where *q*_max_ was the maximum adsorption capacity, *q*_e_ was the bilirubin adsorption amount at different initial concentrations, *C*_e_ was the equilibrium bilirubin concentration, *K*_L_ was the Langmuir isotherm constant, *K*_F_ was the Freundlich constant depicting adsorption capacity, and 1/*n* was the heterogeneity factor indicating adsorption intensity.

The adsorption isotherm fitting results in aqueous solution were summarized in Fig. 4b, c and Table 1. As the fitting coefficients of the Langmuir isotherm being less than 0.5, it failed to describe the adsorption process of the system. The fitting coefficients of the Freundlich isotherm being greater than 0.8, the bilirubin adsorption processes in aqueous solution of all the adsorbents fitted the Freundlich model better. Combined with observations during the adsorption process, it was found that all four adsorbents can induce the aggregation and precipitation of bilirubin in aqueous solutions. Therefore, it is speculated that in bilirubin aqueous solution, the adsorbent not only adsorbs bilirubin, but also causes spontaneous aggregation and precipitation of bilirubin due to breaking the dissolution equilibrium of bilirubin. The fitting results in simulated plasma were summarized in Fig. 4e, f and Table 2, the fitting results show that all four adsorbents have a high matching degree with both models, but the *R*^2^ values fitted to the Freundlich model are higher. According to the fitting results of the Langmuir model, the maximum adsorption capacity of UiO-66-1, UiO-66-NH_2_-1, UiO-66-NH_2_-1.45 and UiO-66-NH_2_-1.9 can be reached 515.4639 mg g^−1^, 143.0615 mg g^−1^, 234.7418 mg g^−1^, 288.1844 mg g^−1^ respectively, which is significantly higher than traditional activated carbon (below 100 mg g^−1^)^9^ and resin material (140.1 mg g^−1^).^8^ In the fitting results of Freundlich model, the 1/*n* values of all samples are less than 1, indicating that the prepared materials are good absorbents for bilirubin.

**Table 1:** Freundlich parameters for bilirubin adsorption on UiO-66-1 and UiO-66-NH_2_ in aqueous solution

Samples	Freundlich isotherm		
	*K*_F_ (dL g^−1^)	1/*n*	*R* ^2^
UiO-66-NH_2_-1	3.8274	1.3133	0.8809
UiO-66-NH_2_-1.45	5.2596	1.3233	0.9300
UiO-66-NH_2_-1.9	6.1636	1.4812	0.9344
UiO-66-1	1.5299	4.8102	0.8683

**Table 2:** Langmuir and Freundlich parameters for bilirubin adsorption on UiO-66-1 and UiO-66-NH_2_ in plasma

Samples	Langmuir isotherm			Freundlich isotherm		
	*K*_L_ (dL mg^−1^)	*q*_max_ (mg g^−1^)	*R* ^2^	*K*_F_ (dL g^−1^)	1/*n*	*R* ^2^
UiO-66-NH_2_-1	0.0151	143.0615	0.9935	2.4691	0.8678	0.9962
UiO-66-NH_2_-1.45	0.0242	234.7418	0.9944	6.1543	0.8620	0.9962
UiO-66-NH_2_-1.9	0.0250	288.1844	0.9907	7.6650	0.8710	0.9985
UiO-66-1	0.0080	515.4639	0.9838	4.3342	0.9405	0.9998

### 3.3 Adsorption kinetics

**Fig. 5:**
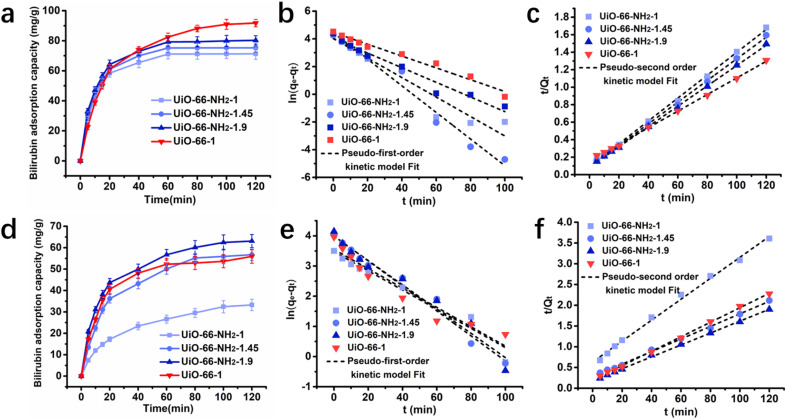
Adsorption kinetics of UiO-66-1, UiO-66-NH_2_-1, UiO-66-NH_2_-1.45 and UiO-66-NH_2_-1.9 (a) curves of bilirubin adsorption amount *versus* time and the fitting results with (b) the pseudo-first-order kinetic model and the (c) pseudo-second-order kinetic model of the samples in aqueous solution; (d) curves of bilirubin adsorption amount *versus* time and the fitting results with (e) the pseudo-first-order kinetic model and the (f) pseudo-second-order kinetic model of the samples in bilirubin loaded simulated plasma.

The adsorption kinetics of UiO-66-1, UiO-66-NH_2_-1, UiO-66-NH_2_-1.45 and UiO-66-NH_2_-1.9 to bilirubin was investigated in aqueous solution and simulated plasma respectively. The initial concentration of bilirubin was both set at 30 mg dL^−1^. In Fig. 5a, it can be seen that in aqueous solution, UiO-66-NH_2_-1, UiO-66-NH_2_-1.45 and UiO-66-NH_2_-1.9 can quickly reach equilibrium within 60 minutes, with equilibrium adsorption capacities of 71.3415 mg g^−1^, 75.2775 mg g^−1^, and 80.3097 mg g^−1^, respectively. UiO-66-1 reached equilibrium after approximately 100 minutes, but its equilibrium adsorption capacity could reach 91.7926 mg g^−1^. In simulated plasma, the adsorption capacity of UiO-66-NH_2_-1, UiO-66-NH_2_-1.45 and UiO-66-NH_2_-1.9 to bilirubin increased with the increase of amino group concentration which shown in Fig. 5d. The equilibrium adsorption capacity of UiO-66-1 is slightly lower than UiO-66-NH_2_-1.45. This indicates that compared to zirconium metal, amino groups have a stronger adsorption effect on albumin bound bilirubin in simulated plasma.

The kinetic behavior was further studied by pseudo-first-order model and pseudo-second-order model. The equations of two adsorption kinetic models are taken in their linear forms as follow:

5 Pseudo-first-order kinetic model: ln(*q*_e_ − *q*_*t*_) = ln *q*_e_ − *k*_1_*t*

6
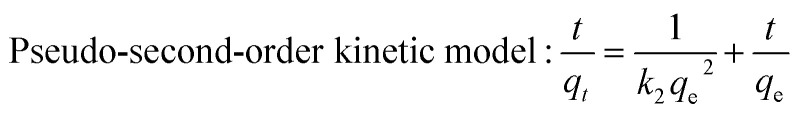
where *q*_*t*_ (mg g^−1^) was the adsorption amount at a given contact time *t* (min), *q*_e_ was the calculated saturation capacity, *k*_1_ and *k*_2_ were the relevant rate constant of the two kinetics models, respectively. By comparing the fitting coefficient (*R*^2^), the *R*^2^ values (*R*^2^ > 0.995) for the pseudo-second-order model were higher than *R*^2^ values (0.921 < *R*^2^ < 0.979) for the pseudo-first-order model. Indicating that all adsorbents in both aqueous solution (Fig. 5b, c and Table 3) and simulated plasma (Fig. 5e, f and Table 4) were more consistent with pseudo-second-order kinetic models.

**Table 3:** Pseudo-first-order and pseudo-second-order kinetic model parameters of the UiO-66-1 and UiO-66-NH_2_ for bilirubin adsorption in aqueous solution

Samples	Experimental	Pseudo-first-order kinetic			Pseudo-second-order kinetic		
	*q*_e_ (mg g^−1^)	*k*_1_ (g mg^−1^ h^−1^)	*R* ^2^	*q*_e_ (mg g^−1^)	*k*_2_ (g mg^−1^ h^−1^)	*R* ^2^	*q*_e_ (mg g^−1^)
UiO-66-NH_2_-1	71.3415	0.0703	0.9213	55.6143	1.862 × 10^3^	0.9988	76.5111
UiO-66-NH_2_-1.45	75.2775	0.0960	0.9736	85.6646	1.783 × 10^3^	0.9989	80.7754
UiO-66-NH_2_-1.9	80.3097	0.0528	0.9619	56.1923	1.565 × 10^3^	0.9993	86.1326
UiO-66-1	91.7926	0.0427	0.9772	86.9289	5.700 × 10^4^	0.9991	105.4852

**Table 4:** Pseudo-first-order and pseudo-second-order kinetic model parameters of UiO-66-1 and UiO-66-NH_2_ for bilirubin adsorption in plasma

Samples	Experimental	Pseudo-first-order kinetic			Pseudo-second-order kinetic		
	*q*_e_ (mg g^−1^)	*k*_1_ (g mg^−1^ h^−1^)	*R* ^2^	*q*_e_ (mg g^−1^)	*k*_2_ (g mg^−1^ h^−1^)	*R* ^2^	*q*_e_ (mg g^−1^)
UiO-66-NH_2_-1	33.2562	0.03317	0.9456	32.3537	1.005 × 10^3^	0.9954	39.6511
UiO-66-NH_2_-1.45	56.7352	0.04143	0.9788	54.5714	8.190 × 10^4^	0.9980	66.0502
UiO-66-NH_2_-1.9	63.1048	0.04021	0.9650	53.8116	1.125 × 10^3^	0.9989	69.6864
UiO-66-1	52.7364	0.03203	0.9232	34.8394	1.593 × 10^3^	0.9989	57.1755

### 3.4 Dynamic adsorption

**Fig. 6:**
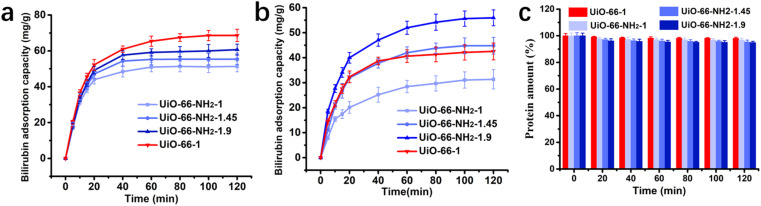
Dynamic adsorption performance of UiO-66-1, UiO-66-NH_2_-1, UiO-66-NH_2_-1.45 and UiO-66-NH_2_-1.9 (a) in aqueous solution and (b) in plasma; (c) total protein contents in plasma during the dynamic adsorption.

To simulate clinical HP process for the treatment of hyperbilirubinemia, a laboratory-made perfusion column connected with peristaltic pump was used to perform dynamic adsorption experiments. Bilirubin aqueous solution and bilirubin loaded simulated plasma circulatory flowed through the perfusion columns with the absorbents. Dynamic adsorption performance of the absorbents is shown in Fig. 6a and b. In the dynamic adsorption process of bilirubin aqueous solution, the adsorbents can quickly reach adsorption equilibrium within 60 minutes, the equilibrium adsorption capacity of UiO-66-NH_2_-1, UiO-66-NH_2_-1.45, UiO-66-NH_2_-1.9 and UiO-66-1 are 51.34 mg g^−1^, 55.38 mg g^−1^, 60.72 mg g^−1^, 68.64 mg g^−1^ respectively. In simulated plasma, the adsorbents can reach adsorption equilibrium within 80 minutes and the equilibrium adsorption capacity of bilirubin is of UiO-66-NH_2_-1, UiO-66-NH_2_-1.45, UiO-66-NH_2_-1.9 and UiO-66-1 are 31.34 mg g^−1^, 44.82 mg g^−1^, 55.97 mg g^−1^ and 42.53 mg g^−1^. The adsorption capacity in dynamic adsorption of adsorbents slightly decreases, but the order and trend of their adsorption capacity are generally the same as in static adsorption. The change in total protein content of simulated plasma after the adsorption process was detected to evaluate the effect of adsorbents on the protein composition in plasma and shown in Fig. 6c. All adsorbents have a minimal adsorption effect on protein components, with only slight changes in protein content after adsorption.

### 3.5 Biological safety testing

**Fig. 7:**
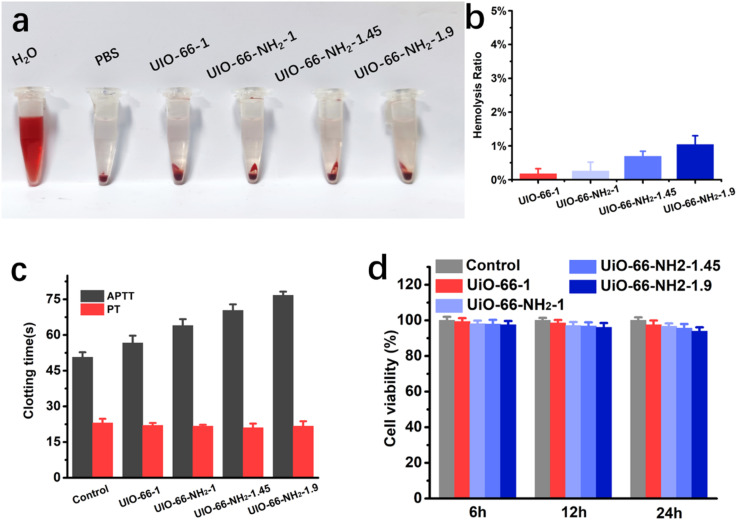
Biocompatibility and blood compatibility tests of UiO-66-1, UiO-66-NH_2_-1, UiO-66-NH_2_-1.45 and UiO-66-NH_2_-1.9. (a) Hemolysis photograph and (b) hemolysis ratio of the hemolysis test. (c) Coagulation time of the samples. (d) Cell viability of 3T3 cells after co-culture with samples for different time.

Due to the adsorbents come into direct contact with blood, their hemocompatibility and cytotoxicity are prerequisites for their practical application. Thus, hemolysis analysis, coagulation times test and cytocompatibility experiments of the adsorbents were conducted. When encountering hemolytic materials, the red blood cell membrane will be destroyed to release hemoglobin, causing the plasma to turn red. It can be seen from Fig. 7a, compared to the control group, all adsorbents have no significant hemolytic effect at a high dose of 3 mg mL^−1^. The hemolysis effect was further quantified by measuring the supernatant absorbance at 541 nm. As shown in Fig. 7b, the hemolysis ratio of UiO-66-1, UiO-66-NH_2_-1, UiO-66-NH_2_-1.45 and UiO-66-NH_2_-1.9 were 0.17%, 0.26%, 0.69% and 1.04% respectively, all significantly lower than the safety level (5%) of biomaterials. Activated partial thromboplastin time (APTT) and prothrombin time (PT) are commonly used indicators to evaluate coagulation abnormalities in both intrinsic and extrinsic pathways. When anticoagulants come into contact with blood, they may bind or react with coagulation factors, leading to an extension of APTT or PT. Fig. 7c shows the APTTs and PTs of UiO-66-1, UiO-66-NH_2_-1, UiO-66-NH_2_-1.45 and UiO-66-NH_2_-1.9, the PTs of all adsorbents were basically consistent with the control group, and APTTs slightly increased with the increase of amino group ratio, this may be attributed to the combination or reaction between the –NH_2_ and the coagulation factors. All adsorbents did not induce any coagulation. 3T3 fibroblasts cells were co-cultured with adsorbents to determine their biocompatibility, cell viability was evaluated using MTT assay (Fig. 7d). Within a 24 hour culture time, all groups of cells maintained high vitality, but with the increase of amino modification degree, cell viability slightly decreased. These results demonstrate that all adsorbents have good blood compatibility, but from the perspective of biocompatibility, the degree of amino modification should not be too high.

## 4. Conclusion

In order to obtain a safe and efficient bilirubin adsorbent, Zr-based MOFs material UiO-66 with high specific surface area and aqueous medium stability was prepared and modified with varying degrees of amination to improve its adsorption capacity. Bilirubin molecules are negatively charged in aqueous solutions, so the unsaturated coordination zirconium on UiO-66-1 has affinity for bilirubin. UiO-66-1 can quickly and effectively adsorb bilirubin and induce aggregation and precipitation of bilirubin molecules in adsorption experiments. This may also be related to the hydrophobicity of bilirubin. In aqueous media, bilirubin is easily agglomerated due to its hydrophobic affinity after being adsorbed on the surface. In simulated plasma, bilirubin binds to albumin, and the adsorption capacity of amino modified UiO-66-NH_2_ is significantly higher than that of unmodified UiO-66-1. Compared to unsaturated coordination zirconium, amino groups have a stronger adsorption effect on albumin bound bilirubin, which may be due to the ability of amino groups to attract carboxyl groups on bilirubin. The adsorption effect of UiO-66-NH_2_ with high degree of amino modification is significantly stronger than that of UiO-66-NH_2_ with low degree of amino modification. The adsorption isotherm curves of UiO-66-1, UiO-66-NH_2_-1, UiO-66-NH_2_-1.45, and UiO-66-NH_2_-1.9 measured were fitted using Langmuir and Freundlich isotherm models, respectively. The fitting results prove that UiO-66 and UiO-66-NH_2_-*X* are all excellent adsorbents for bilirubin. The curve of adsorption capacity *versus* time was measured and fitted using pseudo first order kinetic model and pseudo second order kinetic model. The results showed that the adsorption of bilirubin by all adsorbents are more in line with the pseudo second-order adsorption kinetic model. In dynamic adsorption, the adsorption capacity of adsorbents only slightly decreases compared to static adsorption, and they do not adsorb proteins in plasma. The hemolysis test, coagulation time test, and cytotoxicity test confirmed that all adsorbents have good blood compatibility and biocompatibility. However, when the surface amino density of the adsorbent is too high, the biocompatibility slightly decreases, so the degree of amino modification of the adsorbent should not be too high.

## Conflicts of interest

There are no conflicts to declare.

## Supplementary Material

RA-013-D3RA07212F-s001
