# Supplementary material for: Metal–organic framework (UiO-66 and UiO-66-NH2)-based adsorbents for bilirubin removal used in hemoperfusion
Source: RSC Adv. 2023 Dec 1;13(50):35078–87. doi: 10.1039/d3ra07212f (PMC10691446; doi:10.1039/d3ra07212f)
Supplement: RA-013-D3RA07212F-s001 [file RA-013-D3RA07212F-s001.pdf]

Electronic Supplementary Material (ESI) for RSC Advances.
This journal is © The Royal Society of Chemistry 2023

Metal Organic Framework UiO-66 and UIO-66-NH_2 Based Adsorbents for Bilirubin Removal Used in Hemoperfusion

Yi Liu[1], Zhipeng Yuan[2,*], Yanrong Chen[3,*]

1. Department of traditional Chinese medicine, Jinan Fourth People's Hospital, Jinan, Shandong, PR China.

2. Shandong Key Laboratory for Special Silicon-containing Material, Advanced Materials Institute, Qilu University of Technology (Shandong Academy of Sciences), Jinan, Shandong, PR China.

3. *The Affilited hospital of Shandong university Of traditional chinese medicine, Jinan, Shandong, PR China.*

* for corresponding author.
E-mail address: yuanzp@sdas.org xiaopang-kk@163.com

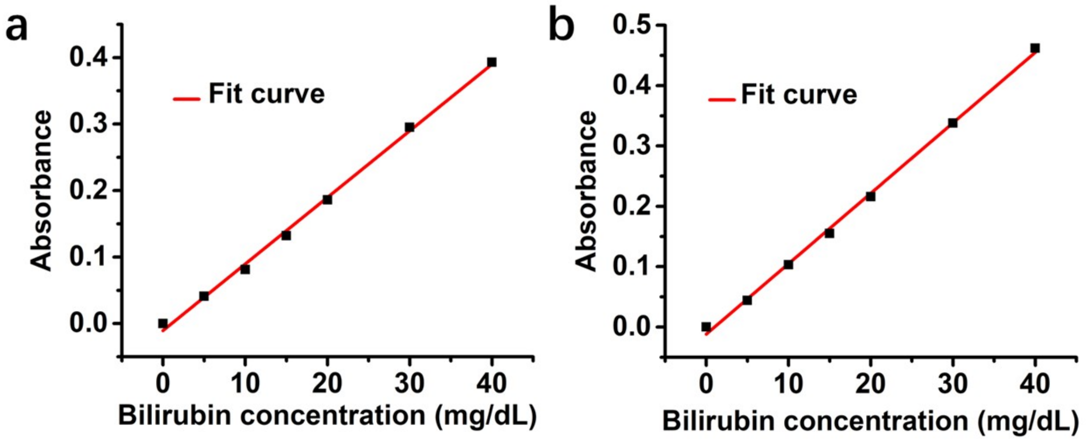

Figure S1 (a) Absorbance at 438 nm of bilirubin aqueous solution with different concentrations and its linear fitting standard curve. (b) Absorbance at 452 nm of bilirubin loaded BSA solution and its linear fitting standard curve. with different bilirubin concentrations.

Table S1 equilibrium adsorption capacity at different initial concentrations of bilirubin in aqueous solution

Samples	Initial concentration of bilirubin in aqueous solution					
	5 mg/dL	10 mg/dL	15 mg/dL	20 mg/dL	30 mg/dL	40 mg/dL
UiO-66-NH_2-1	10.4923	19.8403	30.1863	42.5283	71.2043	102.5416
UiO-66-NH_2-1.45	10.4923	24.4977	35.5090	45.5223	75.1963	104.5376
UiO-66-NH_2-1.9	10.8250	26.1610	39.5010	51.8430	80.1863	110.8583

UiO-66-1	11.1577	27.4917	43.4930	59.8270	91.8297	124.4977

Table S2 equilibrium adsorption capacity at different initial concentrations of bilirubin in plasma

Samples	Initial concentration of bilirubin in plasma					
	5 mg/dL	10 mg/dL	15 mg/dL	20 mg/dL	30 mg/dL	40 mg/dL
UiO-66-NH_2-1	6.3203	12.3977	18.1328	23.6203	33.2467	41.7109
UiO-66-NH_2-1.45	10.2915	20.3236	30.0175	39.0408	56.7236	71.9517
UiO-66-NH_2-1.9	11.3021	22.1952	32.7536	42.9792	63.1139	81.1282
UiO-66-1	9.1811	18.1514	26.9275	35.5877	52.7448	69.0840
